# Dopamine Neuron-Specific Optogenetic Stimulation in Rhesus Macaques

**DOI:** 10.1016/j.cell.2016.08.024

**Published:** 2016-09-08

**Authors:** William R. Stauffer, Armin Lak, Aimei Yang, Melodie Borel, Ole Paulsen, Edward S. Boyden, Wolfram Schultz

**Affiliations:** 1 Department of Physiology, Development and Neuroscience, University of Cambridge, Downing Street, Cambridge CB2 3DY, UK; 2 McGovern Brain Institute, Massachusetts Institute of Technology, Cambridge, MA 02139, USA

## Abstract

Optogenetic studies in mice have revealed new relationships between well-defined neurons and brain functions. However, there are currently no means to achieve the same cell-type specificity in monkeys, which possess an expanded behavioral repertoire and closer anatomical homology to humans. Here, we present a resource for cell-type-specific channelrhodopsin expression in Rhesus monkeys and apply this technique to modulate dopamine activity and monkey choice behavior. These data show that two viral vectors label dopamine neurons with greater than 95% specificity. Infected neurons were activated by light pulses, indicating functional expression. The addition of optical stimulation to reward outcomes promoted the learning of reward-predicting stimuli at the neuronal and behavioral level. Together, these results demonstrate the feasibility of effective and selective stimulation of dopamine neurons in non-human primates and a resource that could be applied to other cell types in the monkey brain.

## Highlights

• Cell-type-specific promoter drives Cre-dependent ChR2 expression in monkey
• Optogenetically activated neurons had dopamine-like features and reward responses
• Dopamine neurons respond strongly to cues predicting optical stimulation
• Monkeys choose predicted optogenetic stimulation over no predicted stimulation

## Graphical Abstract

**Figure:**
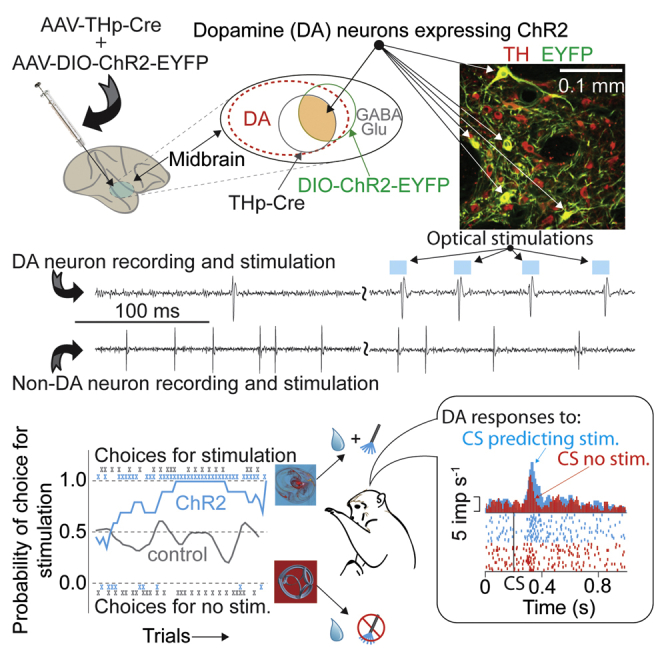

## Teaser

Cell-type specific optogenetic stimulation in Rhesus monkeys allows manipulation of dopamine neurons to demonstrate their role in reward-based learning

## Introduction

Dopamine neurons are involved in many facets of nervous system function and dysfunction (Schultz, 2007, Smith et al., 2014). Numerous studies have suggested that the fast, phasic responses of dopamine neurons code reward prediction errors (Bayer et al., 2007, Bromberg-Martin et al., 2010, Eshel et al., 2015, Fiorillo et al., 2003, Hollerman and Schultz, 1998, Kobayashi and Schultz, 2008, Lak et al., 2014, Ljungberg et al., 1992, Mirenowicz and Schultz, 1996, Nakahara et al., 2004, Schultz et al., 1993, Stauffer et al., 2014, Waelti et al., 2001). Recent optogenetic studies in rodents have demonstrated that dopamine plays a causal role in learning and valuation (Jin and Costa, 2010, Steinberg et al., 2013, Tsai et al., 2009, Witten et al., 2011). However, there is currently no method to apply optogenetics tools specifically to dopamine neurons in monkeys. Thus, detailed circuit-level functionality of dopamine in primate behavior remains unexplored. Monkeys, compared to rodents, possess finer behaviors (Amemori and Graybiel, 2012, Bongard and Nieder, 2010, Stauffer et al., 2014, Stauffer et al., 2015) and greater neuroanatomical homology to humans. Within the dopamine circuit, the anatomical differences are especially pronounced in the mesocortical pathway, which is implicated in working memory, attention, and disease states like schizophrenia (Rolls et al., 2008, Smiley et al., 1994, Smith et al., 2014, Williams and Goldman-Rakic, 1993, Williams and Goldman-Rakic, 1995, Williams and Goldman-Rakic, 1998).

To investigate the circuit-level functionality in a nervous system with high anatomical homology to humans, previous monkey optogenetic studies have employed general purpose (e.g., hSyn, Ef1α) or excitatory-neuron-specific (e.g., CAMKII) promoters (Cavanaugh et al., 2012, Dai et al., 2014, Diester et al., 2011, Galvan et al., 2012, Galvan et al., 2016, Gerits et al., 2012, Han et al., 2009, Jazayeri et al., 2012, Ohayon et al., 2013). These gene promoters are small and can easily fit in the viral vectors commonly used to infect neurons, such as adeno-associated virus (AAV) (Wu et al., 2010). Moreover, these promoters are “strong” promoters; they drive the high levels of gene expression necessary to confer optical sensitivity via ChR2 (Zhang et al., 2010). Nevertheless, these methods do not allow for cell-type-specific manipulation and investigation of the monkey brain function.

Previous methodologies to target specific cell types in wild-type animals have used pathway tracing (Gradinaru et al., 2010, Oguchi et al., 2015) or the construction of synthetic promoters elements (Zalocusky et al., 2016). However, both of these approaches are challenging. Placing anatomically matched injection in monkeys is traditionally very difficult. Likewise, a synthetic promoter would need to be designed for every different cell type. Thus, a general resource would greatly facilitate cell-type-specific optogenetic investigation of monkey behavior.

Here, we set out to express ChR2 exclusively in midbrain dopamine neurons of wild-type Rhesus macaques. We used two viral vectors to accomplish this; the first vector delivered Cre recombinase under the control of a tyrosine hydroxylase (TH) promoter fragment, whereas a second vector delivered a Cre-recombinase-dependent ChR2 construct. The viral vectors were mixed together and injected in the same location. Immunohistological, electrophysiological, and behavioral results demonstrate that the viral vector mixture achieved highly specific expression of ChR2 in dopamine neurons and that optical stimulation activated single neurons and positively affected behavioral readouts of value. By substituting the TH promoter for other neuron-subtype-specific promoters, this optogenetic technique should be amenable to other neuron types in monkey brain.

## Results

### Viral Vectors

**Figure 1:**
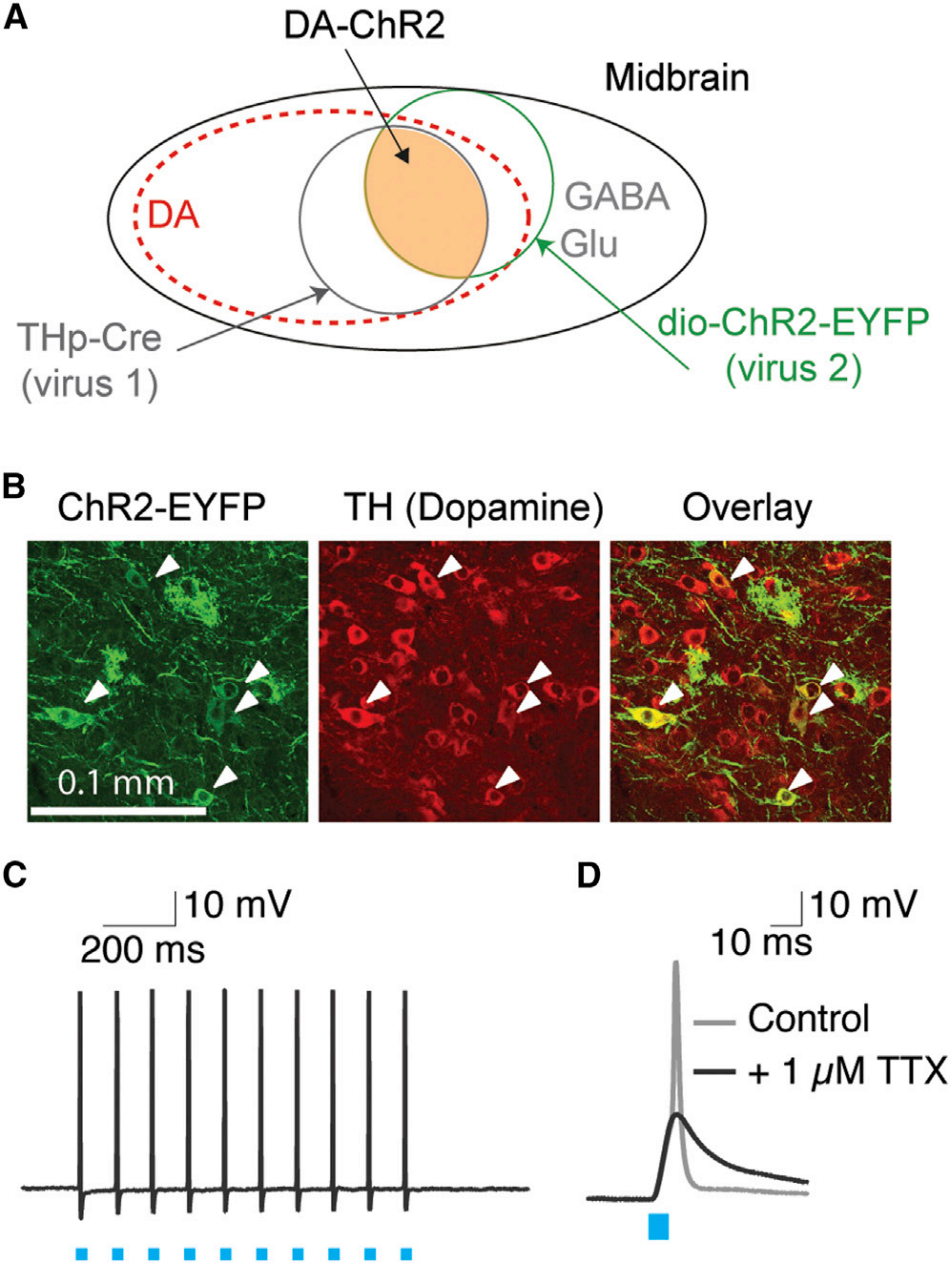
Preliminary Studies Demonstrated Dopamine Neuron-Specific Channelrhodopsin Expression in Wild-Type Mice (A) Schematic diagram of viral infection strategy to gain dopamine-neuron-specific expression of ChR2. Two high-titer viruses (THp-Cre and dio-ChR2-EYFP) were mixed 1:1 for injection. (B) To test the ability of the viral vector combination to induce the expression of ChR2 in dopaminergic neurons of wild-type animals, we injected the vectors into the midbrain of 4-week-old C57BL/6 mice. The expression of ChR2, reported by EYFP, was confirmed a minimum of 2 weeks later using confocal microscopy. The expression of EYFP was compared to the labeling of TH-positive cells in the midbrain. Dopamine neurons that express ChR2 can be seen in yellow in the third panel. (C) To validate the functional efficiency of the vector co-injection, we performed patch-clamp recordings of green neurons in the VTA a minimum of 2 weeks after injection. Brief light pulses repeatedly drove action potentials (APs). In those cells, APs could be triggered by light stimulations at up to 10 Hz. (D) Application of tetrodotoxin (TTX) abolished the spike, but not the light induced potential of the cell in (C), indicating that light flashes were directly driving that neuron, rather than indirectly driving it through synaptic connections. Blue boxes indicate light flashes in (C) and (D).

We injected two viral vectors in a 1:1 mixture to selectively express ChR2 in monkey dopamine neurons and thus distinguish them from the GABAergic and glutamatergic neurons also located in the midbrain (Figure 1A). The first virus used a 300-bp fragment of the 5′ tyrosine hydroxylase (TH) promoter (THp) to express Cre recombinase in dopamine neurons (STAR Methods). The second virus carried a standard Cre-recombinase-dependent ChR2 construct (pAAV5-DIO-Ef1α-ChR2(h134)-EYFP). Optically sensitive dopamine neurons were those that expressed both proteins (Figure 1A, orange shaded region). In preliminary testing, we verified the vector mixture’s expression (Figure 1B) and functionality (Figures 1C and 1D) in wild-type mice and then proceeded to inject these constructs into monkeys’ brain.

### Infection Efficacy

**Figure 2:**
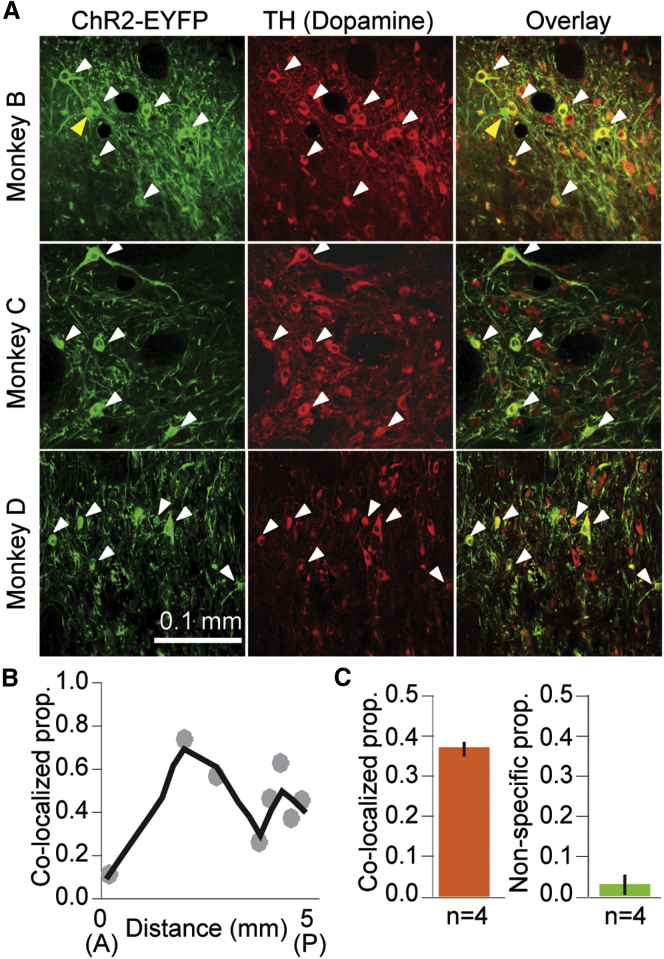
Viral Cocktail Injection into Monkey Midbrain Results in Robust and Highly Specific Expression of ChR2 in Dopamine Neurons (A) Double immunohistochemistry for ChR2-EYFP (green) and TH (red) for three monkeys. White arrows indicate the presence and location of double-labeled cells. The yellow arrow in the top row indicates a non-specific label (a neuron that was positive for ChR2-EYFP, but not TH). These instances were rare and accounted for <5% of the total population. (B) Spatial profile of ChR2-EYFP expression in one animal (monkey A), quantified at multiple coronal sections starting at the anterior midbrain (0) and moving posterior (5). Each data point represents the proportion of dopamine neurons that expressed ChR2-EYFP at each anterior-posterior location (coronal section). Gray line represents the smoothed average of the measured proportion of co-localization. See Figure S1 for a low-magnification view of the expression pattern. (C) Mean proportion of co-localization (left) and specificity (right) for ChR2-EYFP expression across n = 4 animals. Error bars are ± SD across animals. See also Figure S1.

**Figure S1:**
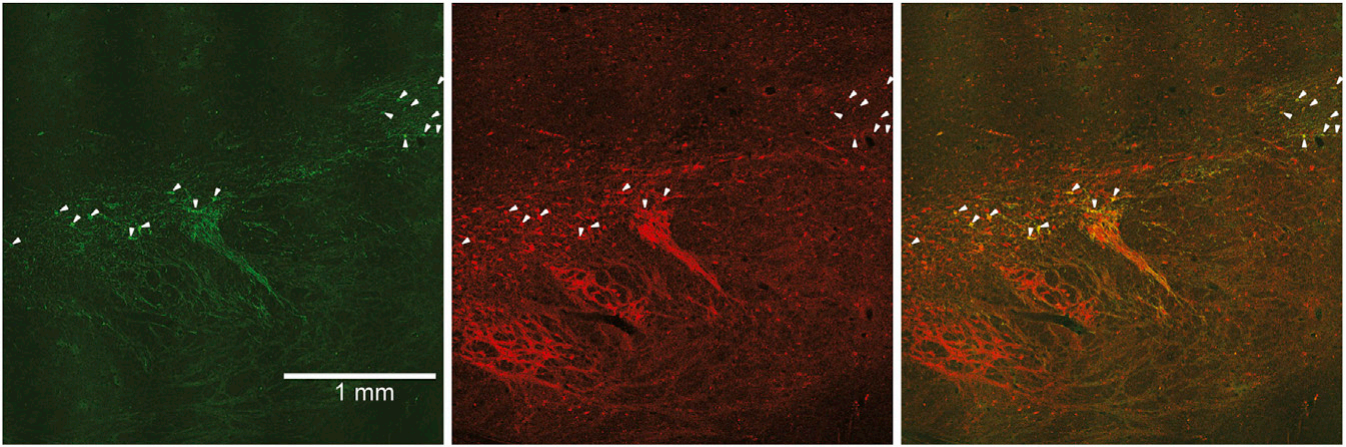
ChR2 Immunoreactivity followed the Irregular Outline of the TH-Positive Cell Bodies in the Monkey Midbrain, Related to Figure 2 Coronal sections through the midbrain show the spatial pattern of overlap between ChR2 immunoreactivity (green, left), TH immunoreactivity (red, middle) and overlay (right). Although quantification of co-localization was done at higher magnification (Figure 2), white arrows indicate some of the colocalized neurons identifiable at this magnification.

Viral vector injections were made alongside electrophysiologically defined monkey dopamine neurons (STAR Methods). To evaluate the efficacy of the viral cocktail for infecting dopamine neurons, we quantified the co-localization of ChR2-EYFP- and TH-immunopositive neurons in four monkeys using high-magnification (20×) images where cell bodies could be easily identified (Figure 2). In all four animals, we observed robust co-localization between ChR2-EYFP- and TH-labeled neurons (Figure 2A, white arrows, 451 of 1,214 counted TH neurons expressed ChR2-EYFP). The specificity of ChR2 expression to dopamine neurons was very high; only a small minority of ChR2-EYFP-positive cells failed to show also TH immunopositivity (Figure 2A, top row, yellow arrow, 21 of 472 ChR2-EYFP-positive neurons). The proportion of infected dopamine neurons approached or exceeded 0.50 on coronal sections near the center of the ventral tegmental area (VTA), where most injections were performed, and fell to as low as 0.10 further away (Figure 2B, monkey A). Averaged across the four animals, the proportion of ChR2-EYFP/TH co-localization was 0.37 ± 0.04 (mean ± SD; Figure 2C, left), whereas the average non-specific labeling was 0.04 ± 0.05 (mean ± SD; Figure 2C, right, n = 4). Moreover, the labeled proportion was remarkably consistent between all subjects (0.39, 0.38, 0.31, and 0.40 in monkeys A, B, C, and D, respectively). The majority of the injections were made within 3–4 mm of midline, and indeed, we did not detect EYFP in the most lateral substantia nigra (7–8 mm from midline). Likewise, EYFP labeling was not detected in the contralateral (un-injected) dopaminergic midbrain. These results indicate that virus cocktail injections resulted in ChR2 expression mostly in dopamine neurons. Lower-magnification images of midbrain provide further support for the vectors’ overall distribution and specificity; the pattern of ChR2-EYFP expression followed the irregular anatomical pattern of TH expression throughout the midbrain (Figure S1). Together, these results demonstrate that the viral cocktail injection resulted in many dopamine neurons expressing ChR2, with very high specificity for this particular neuron type.

### Neurophysiology

**Figure 3:**
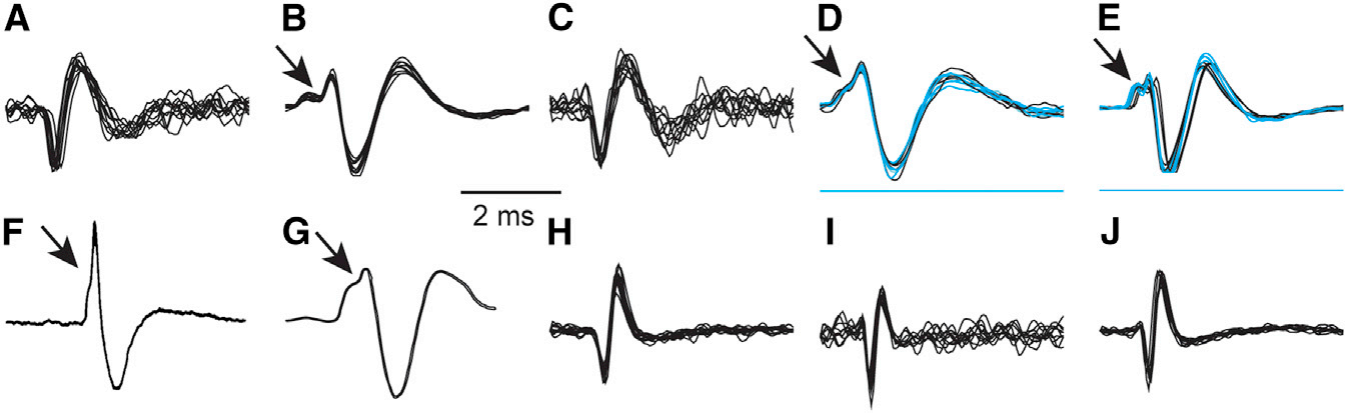
Identification of Dopamine Neurons (A–E) Ten example waveforms from each of five different dopamine neurons. Black waveforms are spontaneous waveforms and blue waveforms are evoked by optical stimulation. Black arrows on (B), (D), and (E) indicate initial segment (IS) breaks commonly seen in dopamine neurons with initial positive deflections. Blue lines in (D) and (E) show the time course of laser pulses. (F and G) Prior studies used apomorphine injections to identify dopamine neurons and consistently demonstrated that apomorphine identified dopamine neurons displayed broad waveforms and prominent IS breaks (black arrows). (F) and (G) were reproduced from Schultz and Romo (1987) and Guyenet and Aghajanian (1978), respectively. (H–J) Ten example waveform from each of three non-dopamine neurons. See Figure S2 for an image of the optrodes used for combined recording and stimulation, and Table S1 for the impulse duration and rate for all recorded neurons. See also Figure S2.

**Figure S2:**
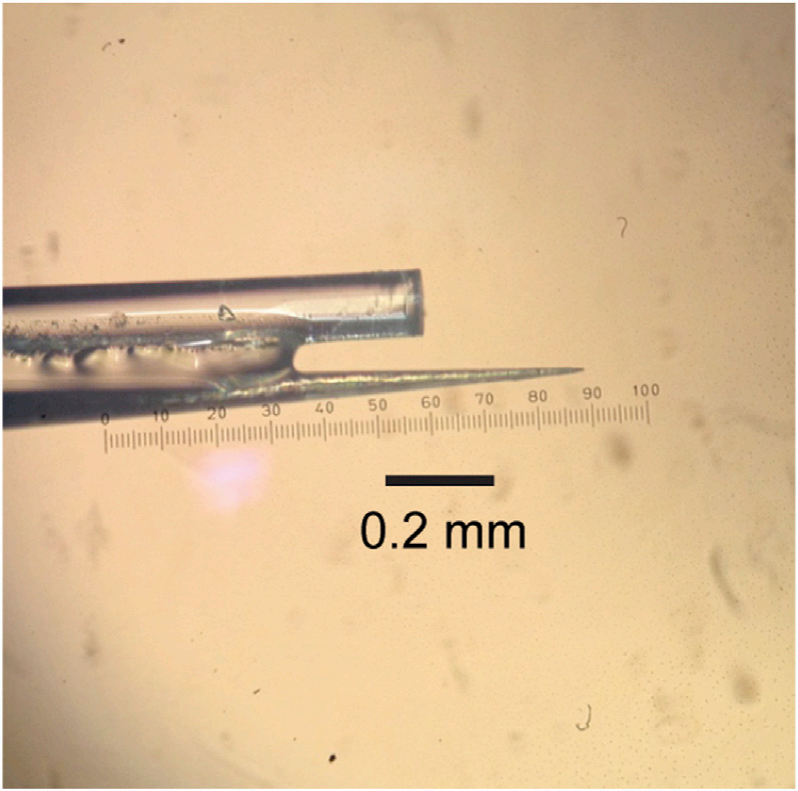
An Example Optrode, Related to Figure 3 Optrodes were constructed by attaching a bare optical fiber to a custom made electrode (STAR Methods). The effective distance between the electrode and fiber tips was between 200 and 500 μm.

To investigate the neurophysiology of ChR2 expression in monkey dopamine neurons, we lowered custom-made electrodes attached to optical fibers (optrodes) into the midbrain of two awake monkeys (C and D) (Figure S2; STAR Methods). We identified putative dopamine neurons (Figures 3A–3E) and non-dopamine neurons (Figures 3H–3J) based on classical extracellular neurophysiological properties, including broad waveforms and low baseline impulse rate (STAR Methods; Table S1), and then delivered pulses of laser light (10 ms) to test for optical sensitivity (Figures 3D and 3E show example waveforms that were optically evoked). Putative dopamine neurons displayed significantly broader action potential waveforms (n = 50, 2.9 ± 0.5 ms [mean ± SD]) and lower baseline impulse rates (n = 50, 5.9 ± 2.3 imp/s [mean ± SD]) compared to non-dopamine neurons (n = 10, duration = 1.7 ± 0.4 ms, impulse rate = 13.7 ± 7.5 imp/s [mean ± SD]) (p < 10^−10^ and 10^−7^ for duration and impulse rate comparisons, respectively, t test). A pronounced initial segment (IS) break was visible in some of the recorded dopamine neurons (black arrows in Figures 3B, 3D, and 3E). An IS break was commonly observed in prior studies where the dopaminergic identity of selected neurons was confirmed by apomorphine injection (Figures 3F and 3G) (Guyenet and Aghajanian, 1978, Schultz and Romo, 1987). Together, these data indicate that the dopamine neurons identified and recorded here were consistent with classically described dopamine neuron properties.

**Figure 4:**
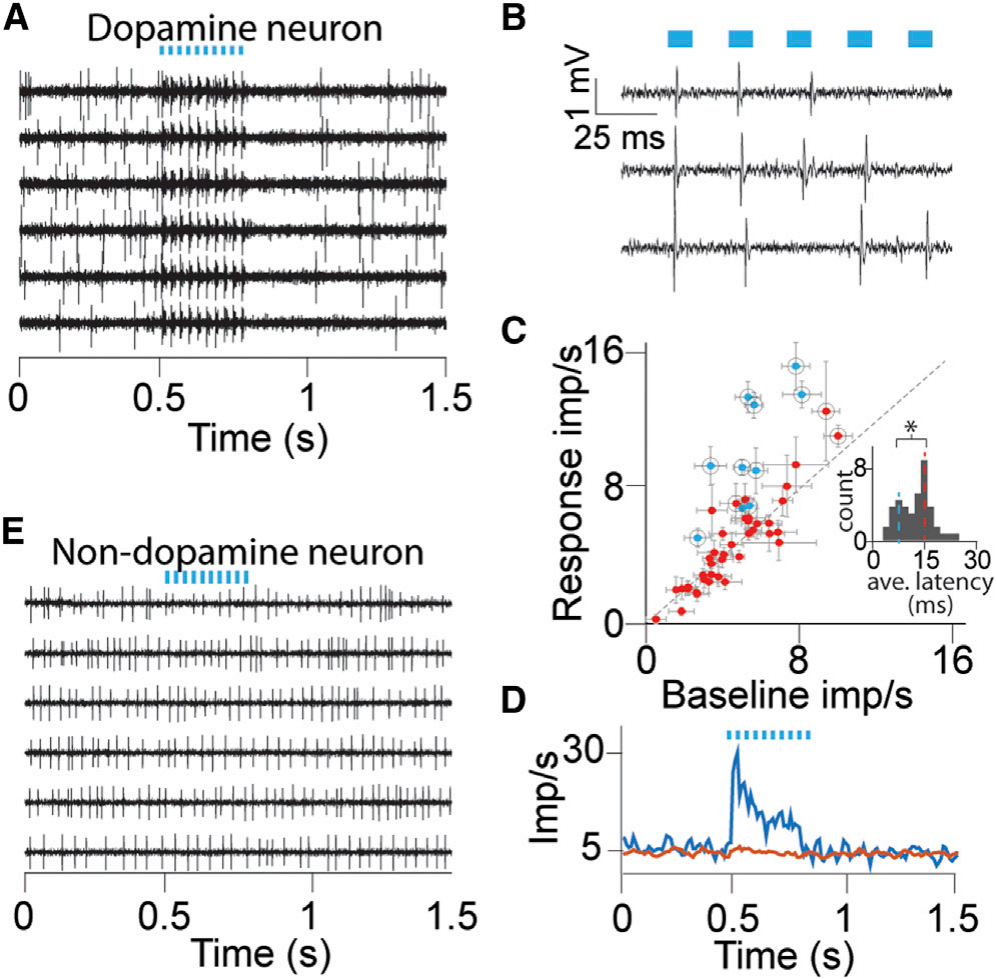
Optogenetic Activation of Monkey Dopamine Neurons (A) Example voltage traces from one dopamine neuron showing light evoked impulses. Blue bars indicate the timing of the laser pulses in (A), (B), (D), and (E). (B) High-time-resolution voltage trace from a second example neuron highlights the widely observed tendency to miss light pulses later in the pulse train. (C) Scatterplot of baseline impulse rate versus optical stimulation response rate for each dopamine neuron. Blue dots represent dopamine neurons that displayed significantly higher impulse rate during optical stimulation, compared to baseline (p < 0.05, Wilcoxon test). Red dots represent neurons that displayed no significant differences. Black circles indicate neurons that formed a cluster with low variability in the latency between optical command and action potential. Error bars are SEM across trials (8–20 trials per conditions, n = 50 dopamine neurons) (inset) Distribution of average (per neuron) latency between timing of optical command and action potential arrival. Blue and red dashed lines indicate the mean latency of all significant and not significant neurons, respectively, as determined by the Wilcoxon test. ^∗^p = 0.03, Hartigan’s dip test. (D) Peri-stimulus time histograms (PSTHs) for two clusters of neurons, identified by k-means clustering of light-onset – action potential latency variability. Blue and red lines derived from 12 and 38 neurons that had smaller and larger latency variances, respectively. (E) Example voltage traces from one non-dopaminergic neuron showing a lack of light evoked impulses.

Pulses of laser (10 ms duration) were delivered while recording neuronal action potentials. In one example dopamine neuron, optical stimulation reliably evoked action potentials on a 1:1 basis; almost every laser pulse caused the neuron to spike (Figure 4A). However, the majority of driven dopamine neurons showed a tendency to miss light pulses delivered later in a pulse train, especially at the high pulse rates we used (Figure 4B, 15–30 ms inter-pulse interval). To distinguish optically sensitive neurons quantitatively, we compared the neural response during the 400-ms light pulse train to the 400 ms of neural activity that immediately preceded it. Out of 50 recorded dopamine neurons, 10 displayed significantly increased impulse rate (Figure 4C, blue dots; p < 0.05, Wilcoxon). The latency of the light-evoked responses also indicated two distinct groups (Figure 4C, inset; p < 0.05, Hartigan’s dip test). Cluster analysis revealed a group of 12 neurons with small response latency variability (K-means clustering with k = 2). The grouping of neurons identified by the clustering algorithm largely overlapped with the neurons that were significant in the Wilcoxon test (Figure 4C, compare blue dots and black circles). Importantly, no neurons displayed a significantly decreased impulse rate in response to light flashes (Figure 4C, red dots; p > 0.05, Wilcoxon test), as might occur if optical stimulation drove local inhibitory neurons. We used the clustering results to divide the neurons into two groups and plotted the resulting population histograms aligned to light pulse train onset (Figure 4D). The two population histograms revealed that only the cluster that contained the short-latency neurons responded to light pulse trains (Figure 4D, blue versus red). Moreover, the optical sensitivity population histogram shows that later light pulses are less effective in evoking spikes in optically sensitive dopamine neurons (Figure 4D, blue line). Finally, light stimulation failed to activate sampled neurons that did not conform to traditional dopamine waveform characteristics (Figure 4E; n = 10). These results demonstrate that the two-virus infection resulted in optically sensitive dopamine neurons and provides electrophysiological support for the immunohistologically observed specificity.

**Figure 5:**
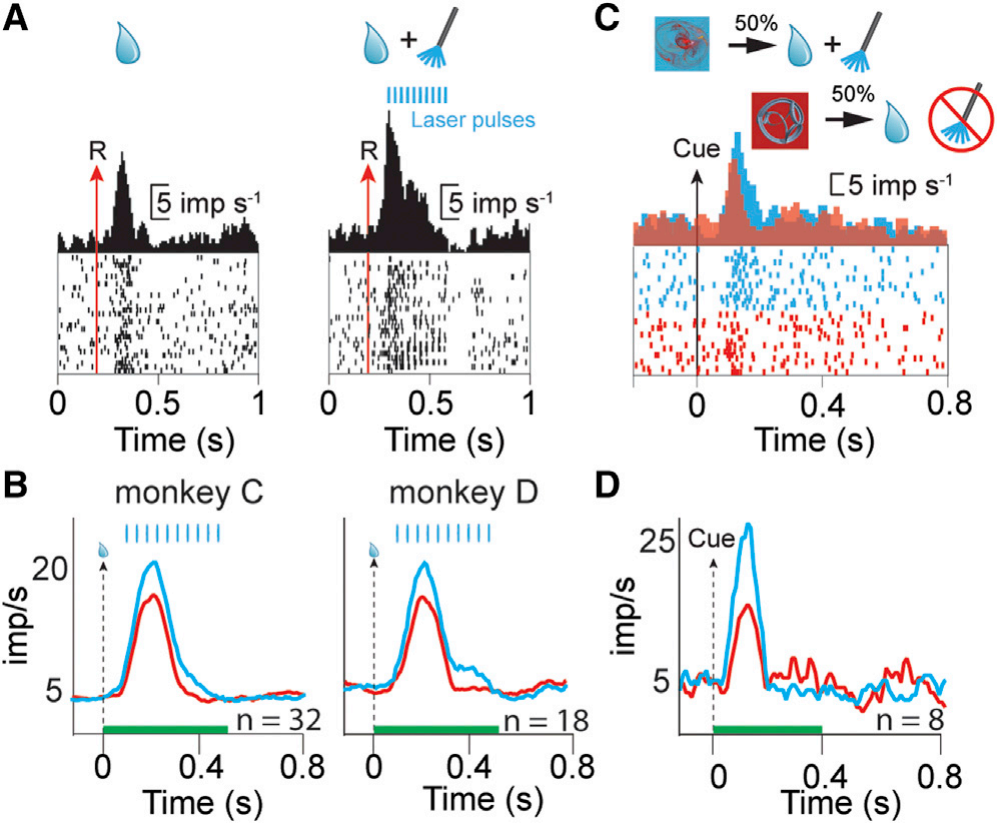
Dopamine-Specific Optogenetic Stimulation Augments Neuronal Response to Reward (A) Optical stimulation facilitated the natural dopamine reward response. Raster plot and PSTH of a dopamine neuron in response to reward (left) and reward plus optical stimulation (right). Blue boxes indicate the laser pulses. (B) Population PSTHs averaged across all neurons (n = 32 and 18 neurons in monkeys C and D, respectively) and aligned to reward alone (red) or reward plus optical stimulation (blue). (C) One cue predicted the delivery of reward plus optical stimulation, whereas a second cue predicted the same reward, delivered alone (top). Blue raster plot and PSTH aligned onto the appearance of cues predicting reward plus optical stimulation (bottom). Red raster plot and PSTH aligned onto the appearance of cues predicting reward alone in the same neuron. (D) Population PSTH averaged across all neurons (n = 8 dopamine neurons from monkey C) and aligned to cue onset. Blue PSTH includes responses to cues predicting reward plus optical stimulation, whereas the red PSTH includes responses to reward alone.

The dopamine reward prediction error response is thought to be a teaching signal, specifically a utility teaching signal (Kobayashi and Schultz, 2008, Lak et al., 2014, Stauffer et al., 2014). Accordingly, optical stimulation of ChR2-expressing dopamine neurons should increase reward subjective value. We compared the neural response to reward plus optical stimulation with the neural response to reward delivered alone. We observed more action potentials following reward plus stimulation than after reward alone (Figure 5A). This positive modulation was strong enough to be significant in both monkeys’ population responses that included all neurons, whether or not they were individually sensitive (Figure 5B; p < 0.05, paired Wilcoxon, n = 32 and 18 in monkeys C and D, respectively). These results demonstrated that the viral manipulation and optical stimulation could significantly augment the natural reward response and suggested that reward plus optical stimulation would have a higher value than the reward delivered alone. Neural evidence for this value difference was observed in the responses recorded from monkey C to conditioned stimuli (CS) that predicted reward plus optical stimulation. CS that predicted reward plus optical stimulation evoked larger neuronal responses than CS that predicted reward alone in single neurons (Figure 5C; p < 0.001 in six of eight neurons, Wilcoxon test) and population responses (Figure 5D; p = 0.015, paired Wilcoxon). These neuronal data predict that the animal will prefer the stimulated option over the non-stimulated option.

### Behavior

**Figure 6:**
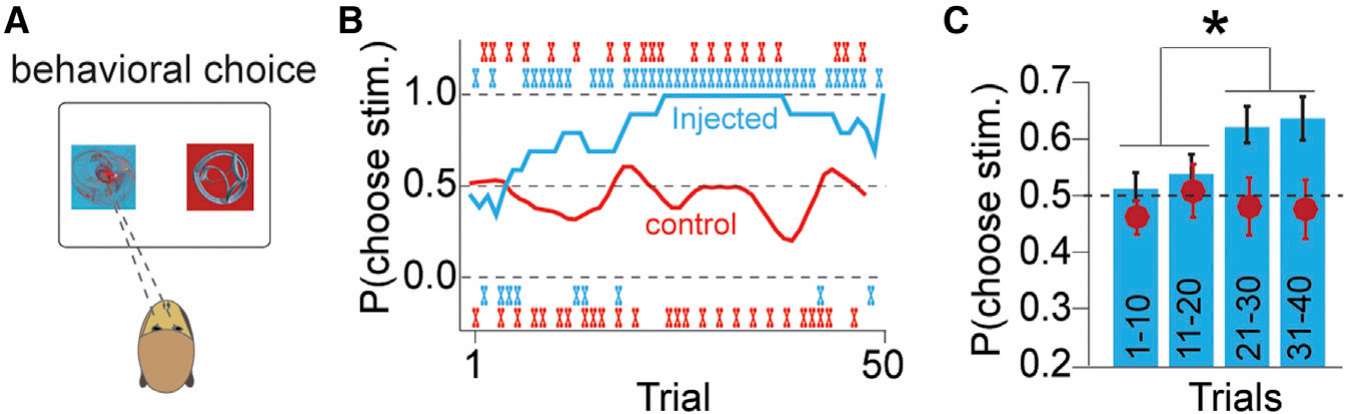
Dopamine-Specific Optogenetic Stimulation Adds Reward Value to a Stimulus (A) Schematic diagram of behavioral setup. Monkeys made gaze-directed choices between two cues that predicted the same liquid reward, but one cue predicted optical stimulation as well (as in Figure 5C). (B) One example learning session with the optical probe in the injected hemisphere (blue line), and one example learning session with the optical probe in the contralateral, non-injected hemisphere (red line). Traces are moving average of ten trials. The “X” marks show the animal’s choices on each trial. The blue (red) X’s indicate choices during the session with the probe in the injected (non-injected) hemisphere. (C) Learning across trials. Blue bars (red dots) represent the average probability of choosing the stimulated option with the probe in the injected (non-injected) hemisphere. Trials were binned into four groups of ten trials and averaged across sessions (n = 43 and 8 CS pairs in monkeys C and D, respectively, for the injected hemisphere; n = 25 and 8 novel CS pairs in monkeys C and D, respectively, for the non-injected hemisphere). Error bars represent ± SEM across sessions. See Figure S3 for similar behavioral data without exogenous reward.

To behaviorally test the prediction that optogenetic stimulation at the time of reward will increase choices for the stimulation-paired reward, we presented the animals with a choice between two naive visual CS; one conditioned stimulus predicted paired reward and optical stimulation, whereas the other conditioned stimulus predicted the same reward delivered alone (Figures 5C, top, and 6A). The animals had to explore both options to learn the values. Within single learning sessions, both monkeys learned to choose the optically reinforced CS (Figure 6B, blue line). Behavioral testing was repeated with new images, which had never before seen before, serving as CS. The monkeys sampled randomly at the start of sessions but learned to prefer the optically stimulated option after ∼10 trials when optical stimulation was delivered to the infected hemisphere (Figure 6C, blue bars; p < 0.03, ANOVA followed by Tukey-Kramer post hoc analysis; n = 43 and 8 CS pairs in monkeys C and D, respectively). Optical stimulation in the contralateral hemisphere had no effect on choice behavior, indicating that non-specific tissue heating sometimes caused by laser flashes was not sufficient to induce a decision bias (Figures 6B, red line, and 6C, red dots; p < 0.9, ANOVA; n = 25 and 8 novel CS pairs in monkeys C and D, respectively). In a separate test, a choice bias toward the stimulated option was observed, even in the absence of exogenous reward (Figure S2; p < 0.01 paired t test, monkey B, n = 8 CS pairs). Together, these data confirm that phasic stimulation of dopamine neurons augments the choice preferences for the option associated with the optogenetic stimulation, thus reflecting an increase in reward value induced by dopamine stimulation.

## Discussion

**Figure S3:**
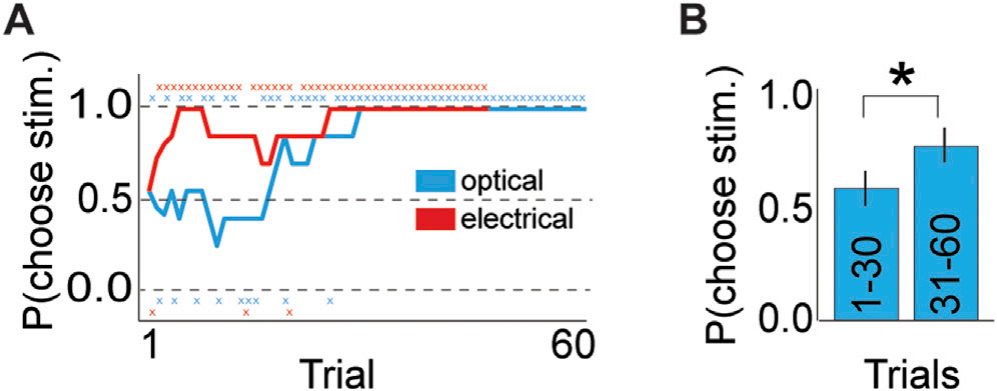
Optical Stimulation Biased Choices in the Absence of Exogenous Reward, Related to Figure 6 The choice experiment illustrated in Figure 6A was repeated in monkey B in the absence of exogenous reward. That is, neither cue predicted delivery of reward (as opposed to both cues predicting reward in the previous test). One cue predicted optical stimulation, the other did not. (A) Blue line shows an example behavioral session with optical stimulation. The monkey started choosing randomly, then slowly but completely became biased to the cue that predicted optical stimulation. The red line shows an example control behavioral session with electrical stimulation (600 μA) in the same location. The monkey quickly developed a preference for the option paired with electrical stimulation. Both lines were smoothed with a 5 point moving average. Blue and red ‘x’ above and below indicate actual trial-by trial choices in the optical and electrical stimulation sessions, respectively. (B) Optical stimulation test was repeated with 8 never before seen cue pairs. We compared the percentage of choices for the stimulated option in the first thirty trials against the same percentage in the last 30 trials. ^∗^ = p < 0.001, t test. Error bars are SEM. across cue pairs.

Here, we demonstrate that a two-virus approach can lead to selective and functional optogenetic labeling of dopamine neurons in wild-type Rhesus macaques. Near the location where the injections were performed, ChR2 expression was seen in >50% of dopamine neurons (Figure 2). The ChR2 expression was highly specific to dopamine neurons, as <5% of ChR2-expressing neurons were not dopaminergic (Figure 2). Moreover, light stimulation drove action potentials in electrophysiologically identified dopamine neurons, but light stimulation elicited no activity in neurons that were not classified as dopamine neurons based on waveform and impulse activity (Figures 3 and 4). As in previous rodent studies, optogenetic stimulation of dopamine neurons during behavior demonstrated that dopamine activity positively influenced behavioral measures of value (Kim et al., 2012, Steinberg et al., 2013, Tsai et al., 2009). This behavioral effect was significant when the optical stimulation was paired with natural juice reward (Figure 6) and in the absence of exogenous reward (Figure S3, but only tested in one animal). Together, these results demonstrate an efficient mechanism for dopamine-neuron-specific optogenetic experimentation in Rhesus macaque brain.

Early on, it was observed that electrical stimulation of dopamine-rich areas provided positive reinforcement (Olds and Milner, 1954). This result was recently observed also in monkeys (Arsenault et al., 2014). Here, dopamine responses to external cues that predicted dopamine optogenetic stimulation were larger than responses to cues that did not predict dopamine optogenetic stimulation (Figure 5). This result suggests that the dopamine reward response is a *neural* teaching signal. Crucially, this signal constitutes a possible neural mechanism for the classically observed behavioral preference for dopamine stimulation.

Many cell-type-specific gene promoters are too large to fit in standard viral backbones. For instance, the entire promoter region of the TH gene is estimated to be ∼7 kb (Kessler et al., 2003). Fragments of cell-type specific gene promoters usually suffer because they are less sensitive and have lower levels of expression, compared to their intact counterparts (Oh et al., 2009, Sohal et al., 2009). Our methods allowed us to use a fragment of the TH promoter. We used the fragment of the TH promoter to drive the expression of Cre recombinase, rather than ChR2 directly. Cre recombinase is an enzyme (Nagy, 2000). Accordingly, it is not consumed during the recombination, and even low Cre recombinase expression can result in robust ChR2 expression. Thus, we sidestepped the issue related to lower levels of expression. Likewise, in our experiments, the specificity did not appear to suffer; it stayed above 95%. The high specificity could be due to the 300 base promoter fragment itself, or it could be due to propensity for the AAV5 serotype to preferentially infect dopamine neurons (McFarland et al., 2009). Larger fragments of the TH promoter delivered by lentivirus (LV) have been shown to drive high levels of GFP expression in monkey dopamine neurons (Lerchner et al., 2014); the use of LV and larger promoter fragments will be an avenue of active research as this technique is further refined.

Recently, controversy has surrounded the electrophysiological identification of dopamine neurons (Ungless and Grace, 2012). Accordingly, there have been renewed questions about whether dopamine neurons preferentially code for information about reward (Fiorillo, 2013, Fiorillo et al., 2013, Mirenowicz and Schultz, 1996) or whether there are distinct sub-populations of dopamine neurons concerned with other variables (Matsumoto and Hikosaka, 2009). Thus, accurate identification of dopamine neurons is a critical issue. Along with apomorphine injections, which selectively silence dopamine neurons (Bunney et al., 1973, Grace and Bunney, 1983, Schultz and Romo, 1987), and juxtacellular labeling (Brischoux et al., 2009), which uses a glass pipette to deliver a dye to recorded neurons, optogenetics has recently been used to identify dopamine neurons and examine their function (Cohen et al., 2012). In the current experiments, every optogenetically identified neuron possessed the broad waveform and low impulse rates classically regarded as identifying dopamine neurons, and all identified neurons responded to reward. On the other hand, none of the neurons with higher impulse rates or shorter waveforms responded to optical stimulation or reward. However, it is important to state that the low numbers of tested neurons in the current study prohibits us from weighing in on this controversy with regard to neuron identification or behavioral function. Nevertheless, future studies can use the resource described here to exhaustively characterize the entire dopamine population.

Perhaps most importantly, our results suggest a framework for attaining cell-type-specific expression in a wide variety of neuron subtypes in the non-human primate brain. Optogenetics has been a powerful toolset to study the functional roles of genetically defined neurons, yet the genetic inaccessibility of non-human primates has limited cell-type-specific studies in these species (Cavanaugh et al., 2012, Dai et al., 2014, Diester et al., 2011, Galvan et al., 2012, Galvan et al., 2016, Gerits et al., 2012, Han et al., 2009, Jazayeri et al., 2012, Ohayon et al., 2013). Our approach enabled the use of a reduced promoter region and sidestepped the efficacy issue associated with using such gene promoters to drive ChR2 directly (Sohal et al., 2009). Small, neuron-subtype-specific promoters have been identified for many neuron types, including D1- and D2-receptor-expressing medium spiny neurons and cholinergic interneurons (Bausero et al., 1993, Minowa et al., 1992, Zalocusky et al., 2016, Zhou et al., 1992). These promoter regions could be swapped with the TH promoter, and these new viruses could be quickly assayed in monkey brain. Moreover, mixing and co-injecting the two viruses simultaneously avoids the difficulty associated with making matched injections into anatomically connected regions, as used in other two-virus approaches (Gradinaru et al., 2010, Oguchi et al., 2015). Thus, these immunohistological, electrophysiological, and behavioral data demonstrate that this two-virus approach works well for specific stimulation of dopamine neurons and suggest a roadmap to gaining neuron subtype specificity in various cell types of the non-human primate brain.

## STAR★Methods

### Key Resources Table

**Table:** 

REAGENT or RESOURCE	SOURCE	IDENTIFIER
**Antibodies**		

Anti-TH antibody, Mouse monoclonal to TH	Millipore	CAT#MAB318
Anti-GFP antibody, Chicken polyclonal to GFP	Abcam	CAT#AB13970; RRID: AB_300798
Alexa Fluor 488 594 donkey anti-mouse antibody	Life Technologies	CAT#A-21203
Alexa Fluor 488 Goat Anti-Chicken antibody	Life Technologies	CAT#A-11039

**Experimental Models: Organisms/Strains**		

Rhesus macaque	The Centre for Macaques (CFM), Defense Science and Technologies Laboratory (DSTL)	N/A
Mouse: wild-type C57BL/6	Harlan, UK	CAT# 057

**Recombinant DNA**		

AAV2/9-rTH-PI-Cre-SV40	Penn Vector Core	N/A
AAV5-Ef1α-DIO-hChR2(H134R)-EYFP-WPRE-pA	UNC Vector Core	N/A

**Software and Algorithms**		

MATLAB	MathWorks	N/A

**Other**		

Optical fiber patch cables 105	Thorlabs	CAT#M15L01
Sharpened optical fibers	Thomas Recording	N/A

### Contact for Reagent and Resource Sharing

Please direct all methodological and resource sharing questions to the corresponding author, William Stauffer (wrs@pitt.edu).

### Experimental Model and Subject Details

Four male Rhesus macaque monkeys (*Macaca mulatta*) were used for these studies (ages: 8.5, 11.8, 10.5, and 11.1 years; weights: 9.1, 13.1,12 and 18.3 kg, respectively). Young adult C57BL/6 mice of either sex (4-8 weeks old; Harlan UK, now Envigo) were housed in polycarbonate cages of 5–10 mice on a 12-h light/dark cycle (7:00 AM–7:00 PM), and had access to food and water ad libitum. The mice were used for preliminary testing of viruses. The Home Office of the United Kingdom approved all experimental protocols and procedures.

### Method Details

#### Surgery and Experimental Setup

A custom-made head holder and recording and stimulating chamber were aseptically implanted under general anesthesia before the experiment. During experiments, animals sat in a primate chair (Crist Instruments) positioned 30 cm from a computer monitor. Eye position was monitored noninvasively using infrared eye tracking (ETL200; ISCAN). Eye data and digital task event signals were sampled at 2 kHz and stored at 200 Hz (eye) or 1 kHz. Liquid reward was delivered by means of a computer controlled solenoid liquid valve (0.004 ml / ms opening time). Custom-made software (MATLAB, MathWorks Inc.) running on a Microsoft Windows XP computer controlled the behavioral tasks as well as the laser.

#### Viral Vectors

We used a novel two-viral vector combination to gain specific optogenetic control of dopamine neurons (Figure 1A top). The first viral vector (pAAV9-TH-Cre-SV40, UPenn Vector Core) delivered Cre recombinase under the control of a 300-base Tyrosine Hydroxylase (TH) promoter sequence. The sequence of the 300 bp fragment was: ctagcggtctcctgtcccacagaataccagccagcccctgccctacgtcgtgcctcgggctgagggtgattcagaggcaggtgcctgtgacagtggatgcaattagatctaatgggacggaggcctttctcgtcgccctcgctccatgcccacccccgcctccctcaggcacagcaggcgtggagaggatgcgcaggaggtaggaggtgggggacccagaggggctttgacgtcagcctggcctttaaagagggcgcctgcctggcgagggctgtggagacagaactcgggaccaccag. The second vector delivered a standard Cre recombinase-dependent ChR2 construct (pAAV5-EF1a-dio-hChR2(H123R)-EYFP-WPRE-pA, UNC Vector Core). The total titer of both viruses was approximately 10^12^ particles (as such, the combination included approximately 5x10^11^ particles of each virus).

#### Virus Injections

The monkey injection coordinates were based upon the location of electrophysiologically identified dopamine neurons. Approximate coordinates were established using an X-ray image. Then, the midbrain was electrophysiologically localized with respect to the somatosensory thalamus. In the anesthetized animal, large cutaneous receptive fields were located on the contralateral limbs by manual manipulation. We advanced the electrode vertically downward and located small receptive fields on ipsilateral and contralateral peri-oral regions, indicating that the electrode tip was located in the ventral posteromedial nucleus of the thalamus (VPM) (Loe et al., 1977). In awake animals, VPM location was confirmed on a daily basis. Liquid reward delivery reliably activated sensory like responses at this depth. Ventral to the VPM, we found ocular pre-motor neurons that fired sharp bursts of action potentials at the onset of saccades. We reliably located putative dopamine neurons approximately 2 mm ventral to the ocular pre-motor responses, and dorsal to and intermixed with tonic eye-position coding neurons. Dopamine neurons were classified based on their well-established electrophysiological signatures, broad waveforms and low background activity (Table S1). Moreover, we verified that these neurons responded to unpredicted reward. As in many previous experiments, we found clear dopamine waveforms and responses 8-13 mm anterior to the intra-aural line, and 2-5 mm lateral to the midline. Our horizontal reference point was arbitrary, taken with respect to the fixed headstage, but we estimate that the dopamine cell bodies were located approximately between 3 and 3.5 cm below the dural surface. In total we injected 65 μl of the viral cocktail into Monkey A, 60 μl into monkey B, 160 μl in monkey C, and 40 μl in Monkey D. In monkey A, the total volume was separated into 65 1 μl injections, whereas in monkeys B-D, we did injections of 20 μl (3, 8 and 2 separate injections in monkey B, C and D respectively). Behavioral and electrophysiological testing started 8 weeks after the final injection.

#### Neuronal Data Recording

Dopamine neurons were localized according to the procedure described above (Virus Injections). Raw data signals were amplified and band-pass filtered between 300 Hz and 5 kHz. Action potentials were isolated on-line using a Bak window discriminator; custom made software running in MATLAB stored the action potential time-stamps and waveforms for later analysis. We recorded a total of 60 neurons (50 dopaminergic and 10 non-dopaminergic) from monkeys C and D.

#### Immunohistochemistry

Sections were cut (50 μm) and stored in sodium azide until staining. Primary antibodies against TH (MAB318, Millipore, used at 1:100) and GFP (AB13970, Abcam, used at 1:200) were combined with Alexa Fluor 594 (A-21203, Life Technologies) and 488 secondary antibodies (A-11039, Life Technologies, both used at 1:1000), respectively. Following a 15 min rinse in PBS, sections were blocked using normal serum, then incubated overnight at 4°C in a solution containing the primary antibodies. Following a 15 min rinse in PBS, sections were incubated overnight at 4°C in a solution containing the secondary antibodies. Sections were mounted using Sigma mounting medium.

#### Optrodes

We used custom made optrodes as well as Thomas optrodes. Custom made optrodes were made from optical fibers (Thorlabs M15L01, 105 μm diameter, NA = 0.22) that were cleaved and then affixed to custom made-glass coated tungsten electrodes using cyanoacrylate. The most effective distances between the optical fiber and the electrode were between 250 and 500 microns. We used these probes to measure the effect of light flashes on dopamine neuron physiology, and a restricted portion of the behavioral tests. Optrodes with a beveled tip were obtained from Thomas recording and used for the majority of the behavioral experiments. These fibers were not paired with electrodes; instead traditional dopamine recordings were performed on separate days to verify the locations were indeed dopamine rich. As a power source, we used a 50 mW, 488 nm laser (SDL-473-050T, Shanghai Dream Lasers Technology Co., Ltd.). We measured the power at the end of the fiber on every recording day using a power meter (Thorlabs, PM100D). We always used the maximum power we could generate (9-15mW). If the maximum power was lower than 9 mW, we didn’t use the fiber.

#### Behavioral Tasks

To test the functionality of the incorporated ChR2, we used a behavioral choice assay. We used a simple preference learning task, which presented the animal with two naive fractal pictures that it had to explore and select between. A central fixation spot appeared on the screen to start the trial. After the animal shifted his gaze to the spot, the two CS were presented at random locations on either side of the spot (sometimes right-left, sometimes top-bottom, and sometimes on the diagonal). The animal had to shift its gaze to one of two cues and hold it there for 0.5 s after which the unchosen cue disappeared. After an additional 1.5 s reward (blackcurrant juice) was delivered. After 50-60 trials, the two fractals were replaced with another two naive fractals.

#### Quantification and Statistical Analysis

Statistical values including the exact n, the definition of center, dispersion and precision measures and statistical significance are reported in the Figure Legends and in the main text. ANOVA was used where appropriate (i.e., multiple comparisons), and data were judged to be statistically significant when p < 0.05.

##### (1) Immunohistochemistry

We counted all TH and GFP immunopositive cells and the number of co-localized neurons on coronal sections at different anterior-posterior positions. We then added together all the neurons counted in a given animal. The proportion of co-localized cells in each animal was expressed as the total number of co-localized cells on all sections counted / the total number of TH positive cells on all sections counted. Likewise, the proportion of non-specific labeling was reported as the total number of GFP+/TH− cells on all sections counted / the total number of GFP+ positive cells on all sections counted. Means and standard deviations were calculated across animals, and all statistics were done across animals (n = 4 animals).

##### (2) Neuronal data

To evaluate light-evoked neuronal responses, we compared the number of spikes that occurred during the laser pulse train, and the number of spikes in an equally-sized time window before the pulse train (Wilcoxon sign-rank test, p value threshold was 0.05). Significantly modulated neurons are indicated in blue in Figure 4C. To perform the response latency analysis, we isolated every light flash and measured the time to subsequent spike. We used Hartigan’s dip test on the distribution of average latencies to determine whether there was more than one population of dopamine neurons. This test was significant, indicating two distinct populations of latencies. Therefore, we used K-means clustering with 2 clusters on the standard deviations of the individual neuron latencies. The members of the cluster of neurons that displayed small latency variability are circled in Figure 4C, and are used to compute the blue Peri-Stimulus Time Histogram (PSTH) in Figure 4D. PSTHs were computed using 10 ms bins, and smoothed with a 100 ms sliding window. For population PSTHs, a PSTH was computed for each neuron, and then averages were taken across individual PSTHs.

##### (3) Behavior

Individual behavioral sessions were smoothed with a ten-step moving average filter for display (Figure 6B). For statistical analysis of choice behavior, we binned choices into 10 trial blocks and computed the choice frequency for the stimulated options (Figure 6C, mean ± SEM). We used ANOVA with Tukey-Kramer posthoc analysis to test for significance.

### Data and Software Availability

#### Software

MATLAB was used for all data collection and analysis.

## Author Contributions

W.R.S., A.L., E.S.B., and W.S. designed all experiments. W.R.S., A.Y., and E.S.B. developed viral vectors. M.B. and O.P. carried out rodent experiments. W.R.S. and A.L. performed monkey experiments. W.R.S., A.L., and W.S. performed data analyses and wrote the paper.
